# Bacteriological profile, antibiotic susceptibility and factors associated with neonatal Septicaemia at Kilembe mines hospital, Kasese District Western Uganda

**DOI:** 10.1186/s12866-021-02367-z

**Published:** 2021-11-04

**Authors:** Henry Zamarano, Benson Musinguzi, Immaculate Kabajulizi, Godfrey Manirakiza, Walker Guti, Ivan Muhwezi, Ayan Ahmed Hussein, Agnes Baweera, Boaz Kabahinda, Herbert Itabangi, Joel Bazira, Taseera Kabanda

**Affiliations:** 1 Department of Microbiology, Faculty of Medicine, Mbarara University of Science and Technology, P.O Box 1410, Mbarara, Uganda; 2 Department of Medical Laboratory Science, Faculty of Health Sciences, Muni University, P.O. Box 725, Arua, Uganda; 3 Department of Microbiology and Immunology, College of Health, Medicine and Life Sciences, King Ceasor University, P.O. Box 88, Kampala, Uganda; 4 Department of Microbiology and Immunology, Faculty of Health Sciences, Busitema University, P.O Box 1460, Mbale, Uganda

**Keywords:** Neonatal, Septicaemia, Bacteriological, Antibiotic

## Abstract

**Introduction:**

Neonatal septicaemia is one of the most common leading causes of neonatal morbidity and mortality in developing countries. It is estimated to affect more than 30 million people worldwide annually, potentially leading to 6 million deaths.

**Objective(s):**

To determine the prevalence, bacteriological profile, antibiotic susceptibility and factors associated with neonatal septicaemia among neonates suspected to sepsis at Kilembe mines hospital.

**Methods:**

We conducted a descriptive cross-sectional study, where purposive sampling technique was used and blood was drawn from 122 neonates suspected to sepsis attending Kilembe Mines Hospital during the period (July to November 2020). Specimens were inoculated in Brain heart infusion broth, transported to Fortportal Regional Referral Hospital, plated daily up to 7 days on blood, chocolate, MacConkey agar and incubated in aerobic and 5% carbondioxide. Pure colonies were identified by Gram stain, biochemical tests and antibiotic sensitivities obtained by Kirby Bauer disc diffusion method. Associations were tested using Chi square with Fisher’s exact or Yates correction tests where necessary and statistical significance was set at *P* < 0.05. Stata (version 14) used for statistical analysis.

**Results:**

Blood cultures were positive in 59.0% cases with 55.5% male and 44.4% female. EOS was present in 56.9% and LOS 43.1% of the cases. Gram negative (56.9%) organisms were most implicated with neonatal septicaemia than Gram positives ones (43.1%). Gram positive organisms exhibited better susceptibility to amikacin, linezolid and vancomycin but more resistant to ampicillin and gentamicin. Of the aminoglycosides, amikacin exhibited a verge over netilmicin and gentamicin against Gram negative isolates. Risk factors of neonatal septicaemia were mother’s age of ≥25 years, employed mothers, tertiary-level of education, SVD, ANC attendance of ≥4 times, UTI during pregnancy, PROMS, foul Smelling liquor, urban residence, neonatal birth weight of ≥2500 g, Apgar score 1st and 5th min ≥6 and resuscitation.

**Conclusion:**

Multi-drug resistant organisms were isolated. Therefore caution is required in selection of antibiotic therapy and avoid empirical treatment.

## Background

Neonatal septicaemia is a blood infection that occurs in the first 4 weeks of life renowned by a *positive* blood *culture* [1]. Septicaemia in neonates can lead to sepsis which is a clinical syndrome of bacteremia characterized by systemic signs and symptoms of infection in less than 28 days of life, manifested by isolation of bacterial pathogens which gain access into the blood stream causing Early onset septicaemia (EOS) that occurs in the first 72 h of life or Late onset septicaemia (LOS) that occurs beyond 72 h of life [2].

The most commonly isolated bacterial organisms causing neonatal septicaemia include: *Staphylococcus aureus*, *Escherichia coli*, and *Group B Streptococci* [3]. Infections, prematurity, birth asphyxia, low birth weight and other factors like type of delivery, contribute to incidences of neonatal septicaemia [4]. *Staphylococcus aureus* septicemia is associated with a mortality rate of 15 to 30%. 15 to 30% mortality rate is associated with *Staphylococcus aureus* septicemia mainly by methicillin-resistant *S. aureus* (MRSA) [5] Extended-spectrum beta-lactamases (ESBL)-producing Enterobacteriaceae are life threating and they are largely associated with blood stream infections in neonates. These render carbapenems a mainstay in treatment of septicaemia caused by ESBL-producing pathogens [6].

Globally, the burden for neonatal septicaemia increased from 36% in 1990 to 43% deaths in 2011 [7] and neonatal sepsis is 2202 (95% CI: 1099–4360) per 100,000 live births with 11 to 19% mortality [8]. Infections leading to sepsis are responsible for about one-fifth of the world’s annual 2.7 million neonatal deaths, in South Asia and sub-Saharan Africa (developing countries), it is about 98% of all neonatal deaths [9]. The Incidence of neonatal septcaemia is around 54.9 per 1000 live births for inborn infants with a mortality rate of 19% of the fatalities attributed to Gram-negative organisms which are mainly susceptible to gentamicin, ceftriazone and cefuroxime [10].

The neonatal mortality rate (NMR) in low income countries like Uganda is 9 times higher than the average NMR in high-income countries with 3·0 deaths per 1000 livebirths. The sub-Sahara Africa accounts for 19% and the East and South African regions contributing to around 18% of neonatal deaths [11] with Uganda having an under estimated rate of 29 deaths per 1000 live births [12].

Several factors have been found to put neonates at risk of acquiring septicaemia. These factors range from sex, history of convulsions, hypoglycaemia, lack of antenatal care, late onset sepsis, umbilical pus discharge [4], preterm labor, premature rupture of membranes (PROM), intra partum, fever and neonatal low birth weight [13]. Neonates from low socio-economic status or rural backgroungs have increased risks of acquiring or developing septicaemia due to exposure to unhygienic conditions [12]. There are also pregnant women who don’t attend antenatal care at the health facilities therefore missing an opportunity of screening and treatment for infections that could be passed onto their neonates. The use of traditional birth attendants and delivering at home has also been associated with higher risk of newborns developing septicaemia especially in developing countries [14].

In Uganda, NMR is 27 deaths per 1000 live births [15]. This differs between rural and uban areas as well as the poor and rich house holds forexample, in rural areas, the NMR is 30 deaths per 1000 live births and 31 deaths per 1000 live births in urban areas and among the poor households is 26 neonatal deaths per 1000 live births, compared to 34 deaths per 1000 live births among the rich households [16].

The nature of bacteria responsible for neonatal septicaemia also vary from time to time in different settings and even from region to region. It can even vary from hospital to hospital in the same city [17].

At Kilembe mines hospital, a review of data for the year 2018 revealed that 117 neonates were admitted and managed for septicaemia, of these, 2 died giving a death rate of 1.7% deaths according Health Management Information Systems (HMIS) 107 year 2018/2019. Some cases were not documented following comparision of records from the daily requesters and the HMIS 107. Kilembe mines hospital being the only general hospital around Kasese town, it frequently receives neonates with complications as well as complicated pregnancies than the surrounding low level health facilities yet this facility lacked a microbiology laboratory with capacity to conduct blood cultures hence promoting erratic use of unbiotics on neonates without any positive blood culture proven results.

Thus, in order to reduce morbidity and mortality due to neonatal septicaemia, there should be deliberate efforts to identify the bacteria responsible for neonatal septicaemia and their susceptibility to the commonly available antibiotics. The study aimed at providing information on bacteriological profile, antibiotics susceptibility and factors associated with neonatal septicaemia at Kilembe mines hospital.

## Methods

### Study design

This was a descriptive cross-sectional study where purposive sampling technique was used to identify the 122 neonates suspected to sepsis attending Kilembe Mines Hospital in Southwestern Uganda between July and November 2020.

### Inclusion criteria

i. All neonates with clinical signs of sepsis attending Kilembe mines hospital during the study period.

### Exclusion criteria

i. Neonates with a history of antibiotic therapy within 2 days.
ii. Neonates who were on intermittent presumptive treatment (to avoid false negative results).

### Data collection

Parents or guardians who consented for their neonates and healthy workers who participated in the study were subjected to an interviewer guided questionnaire to capture information on social demographics and clinical data.

Qualitative data was gathered using a questionnaire from the mothers or guardians of the neonates at Kilembe mines hospital.

### Sample collection and processing

All blood cultures were collected before starting any antibiotic therapy.

Approximately 2 ml of venous blood were obtained from neonates by a doctor after thorough disinfection of the patient’s skin for approximately 2 min with 70% alcohol and allowed to dry before taking blood. One millilitre of blood was collected in each of two bottles containing brain heart infusion (BHI) in a ratio of blood: BHI of 1:10 and taken to Fortportal regional referral hospital microbiology laboratory. Each bottle was incubated at 37 °C for 24 h, these were examined for visible growth and Gram staining was done. Subcultures were plated daily up to 7 days on blood agar, MacConkey agar incubated in aerobic and chocolate agar in 5% carbondioxide conditions. Pure colonies were identified by Gram staining, biochemical tests and antibiotic sensitivities were obtained [18]. For a blood cultures that showed no visible growth and negative on Gram staining, three subcultures were done on blood agar, Chocolate and MacConkey agar and observed for a maximum of 7 days before being discarded as negative if no growth. Discarded all culture bottles with mixed growth (those with more than 2 types of bacteria). Also fom the chocolate agar inoculations, subcultures were made in mannitol salt agar, bacterial colonies showing typical characteristics of *S. aureus* such as golden yellow color colonies on mannitol salt agar were subjected to gram staining, catalase test, and DNase test. Mannitol-fermenting, gram-positive bacteria appearing as grape-like clusters and exhibiting catalase positivity were subcultured in DNase agar and incubated for 24 h at 37 °C. DNase agar plates were subsequently flooded with HCl (1 N) and for those Isolates that exhibited the ability to hydrolyze DNA were identified as *S. aureus* [19].

*Screening for* extended spectrum beta-lactamases (ESBLs) production was done using Cefotaxime (30 μg), Ceftazidime (30 μg) and Ceftriaxone (10 μg) discs. Where the organism was found to be resistant, was classified as screening positive. Double disc synergy was also used to confirm ESBL production. This involved use of a single disc of Cefotaxime 30 μg or Ceftriaxone (10 μg) and a combination disc (Cefotaxime (30 μg*)/Clavulanic acid and* Ceftazidime (30 μg)/ *Clavulanic acid). An increase in the zone diameter of ≥ 5 mm between the single disc and combined disc was considered as ESBL positive in accordance to Clinical and Laboratory Standard Institute 2020 guidelines* [19, 20]*.*

The disk diffusion method adopted from the Clinical laboratory Institute was used to assess the antimicrobial susceptibility of all the isolates [21]. The study used the commonly used antimicrobial drugs in the Uganda national treatment guidelines for neonatal septicaemia such as β-lactam/βlactamase inhibitor (amoxicillin/clavulanic acid), cephalosporins (ceftriaxone, cefotaxime), carbapenem (imipenem), aminopenicillin (ampicillin), aminoglycosides (amikacin, gentamicin) and cotrimoxazole for Gram negative organisms, β-lactam/β-lactamase inhibitor (amoxicillin/clavulanic acid), cepholosporins (cefoxitin, cefotaxime, ceftriaxone), aminoglycosides (amikacin, gentamicin, netilmicin), aminopenicillin (ampicillin), vancomycin, linezolid and cotrimoxazole for Gram positives organisims to assess susceptibility of the isolates [22].

### Quality control and testing procedures

Every new batch of culture media was incubated at 37 °C overnight to check sterility. Reference strains *ie E. coli* American Type Culture Collection) (ATCC) 25,922, *K. pneumoniae* ATCC 700603, *Pseudomonas aeruginosa* ATCC 27853, *Enterococcus faecalis* ATCC 29212 and *Staphylococcus aureus* ATCC 25923 were used as quality control strains for biochemical and antimicrobial susceptibility testing [23]. This study used commercially prepared blood culture media, stains, drug sensitivity discs and biochemical/Analytical profile index (API) [24].

### Statistical analysis

The raw data was entered into excel spread sheets and later imported to Stata (version 14) for statistical analysis.

The characteristics of the study samples were described using measures of central tendency like median, mean and range. Different species of bacteria were sorted out and proportions of each isolated bacterium was compared to assess the most prevalent species involved in neonatal septicaemia. Diameters of the zones of inhibition (mm) was used to report the antibacterial activity. Associations were tested using Chi square with Fisher’s exact or Yates correction tests where necessary and evaluations carried out at 95% confidence level (95% CI) and values of (*p* < 0.05) were regarded as significant. Results were presented in form of tables and graphs.

## Results

### Recruitment procedure: recruitment and neonatal clinical characteristics/features

In this study, 141 neonates were screened but 122 neonates met the inclusion criteria and these were enrolled into the study. The rationale of the 19 neonates that were excluded. Of the 122 neonates, 3 presented with Irritability, 2 with Abdominal distension, 15 with Umbilical redness extending to the skin or infection, 5 with hypothermia/ feeling cold, 4 with Vomiting, 16 with jaundice, 3 with difficult to wake up/lethargy, 10 with temperature ≥ 37.5 °C or felt hot to touch, 14 with hypothermia (≤35.0 °C) or felt cold on touching, 3 with Convulsions, 30 with respiratory distress and 17 not able to feed and not able to attach to the breast or suck.

### Neonatal characteristics and demographics

This study recruit 69(57%) neonates between the age of 0–72 h and 53(43%) neonates between the age of 72–4 weeks. 53(43%) of these were female 69(57%) and 79(65/%) male.

### Prevalence of neonatal septicaemia

**Fig. 1:**
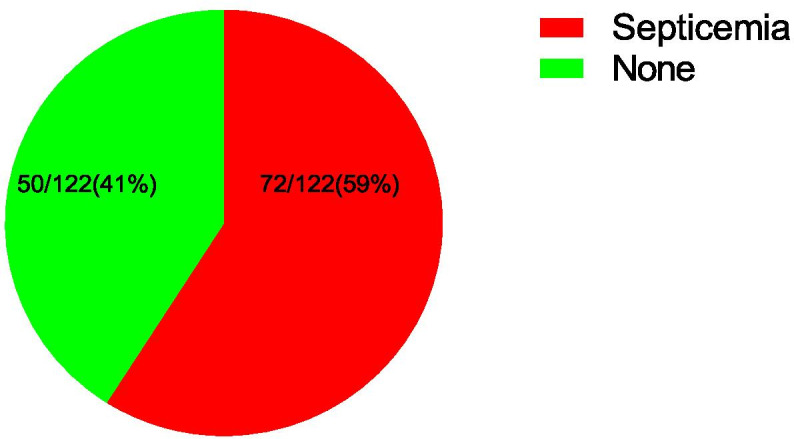
Prevalence of neonatal septicaemia

Out of the 122 participants, 72 (59%) has septicaemia while 50(41%) had no septicaemia making overall prevalence of neonatal septicaemia among neonates seeking medical services at Kilembe mines hospital as 59% as shown in Fig. 1 below.

Of the 122 participants, 69 (56.6%) were male and 53(43.4%) female. 69(56.6%) Presented with early on set septicaemia while 53 (43.4%) were of late on set septicaemia.

### Bacteria responsible for neonatal septicaemia

There were 11 Bacteria responsible for neonatal septicaemia identified from 122 study participants was atotal of 72 isolates. *Streptococcus agalactiae* were more common among the neonate with a percentage of 21% followed by *S. aureus* 19% and others. Among these, 41/72 (56.9%) cases were of early onset septicemia and 31/72 (43.1%) cases were of late onset septicemia. *Prevalence of maximum* extended spectrum beta-lactamase *(ESBL) positivity was observed for Klebsiella pneumoniae (40%), followed by Escherichi coli* (25%), *Enterobacter aerogenes* (14.3%) and *Acinetobacter spp* (12.5%) amongst the rest of the organisms that had 0 prevalence. as shown in the Table 1 below.

**Table 1:** Bacteria responsible for neonatal septicaemia

Organism	EOS	LOS	Frequency	%	ESBL Positive	
					No.	%
**Gram-positiveorganisms**						
*Staphylococcus aureus*	8	6	14	19	0	0
*Streptococcus agalactiae*	9	6	15	21	0	0
*Enterococcus.spp.*	1	0	1	1.4	0	0
*Viridans streptococci*	1	0	1	1.4	0	0
**Gram-negative organisms**						
*Pseudomonas aeruginos*	4	2	6	8	0	0
*Citrobacter freundii*	1	0	1	1.4	0	0
*Escherichia coli*	3	5	8	11	2	25
*Enterobacter aerogenes*	3	4	7	10	1	14.3
*Klebsiella pneumoniae*	6	4	10	14	4	40
*Acinetobacter spp*	5	3	8	11	1	12.5
*Proteus mirabilis*	0	1	1	1.4	0	0
**Total**	41	31	72	99.6	8	91.8

### Susceptibility pattern of commonly used antimicrobial agents in the treatment of neonatal septicaemia

The analysis of drug resistance pattern showed that, among Gram-negative isolates, maximum numbers 92.9% were resistant to ampicillin, Cefoxitin (76.7%), Cotrimoxazole (70.6%), Ceftriaxone (64.3%), Netilmicin (59.5%), Gentamicin (58.9%), Amikacin (53.3), Amoxycalvulinic acid (46.9%), Cefotaxime (45.5%), Linezolid (33.3%) and lowest to imipenem (25.5%). Among Gram-positive isolates, high resistance was seen to ampicillin (100%), Gentamicin (80.9%), Ceftriaxone (72.3%), Cotrimoxazole (69.1%), Amoxycalvulinic acid (50.9%), Cefoxitin (41.9%) and Amikacin (25.4%) as shown in Table 2 below. However, among the Gram negative oganisms, there was higher sensitivity to Ampicillin (74.5%) followed by Linezolid (55,7%), Amoxyl/calvulinic acid (53.0%), Cefoxitin (51.7%), Amikacin (46.5%), Gentamicin (41.0%), Netilmicin (36..4%), Ceftriaxone (35.7%), Cotrimoxazole (51.7%), Cefoxitin (20.4%) and lastly to Ampicillin (7.0%). Among Gram-positive isolates, there was more sentivity to Vancomycin (100%), Netilmicin (91.7%), Amikacin (89.4%), Cefotaxime (77.6%), Cefoxitin (58.1%), Amoxyl/calvulinic acid (49.0%), Cotrimoxazole (30.9%), Ceftriaxone (27.7%), Gentamicin (24.0%) and 0% sentivity to Ampicillin as shown in Table 3 below.

**Table 2:** Susceptibility pattern of commonly used antimicrobial agents in the treatment of Gram positive isolates causing neonatal septicaemia

Sn	Antibiotics (disk content in μg)	Gram-positiveorganisms							
		*Staphylococcus aureus*		*Streptococcus agalactiae*		*Enterococcus.spp.*		*Viridans streptococci*	
		S (%)	R (%)	S (%)	R (%)	S (%)	R (%)	S (%)	R (%)
1	Ampicillin(10)	0	100	0	100	0	100	0	100
2	Gentamicin(10)	42.9	57.1	33.3	66.6	0	100	0	100
3	Amoxyl/calvulinic acid(20/10)	42.9	57.1	53.3	46.6	100	0	0	100
4	Ceftriaxone(10)	64.3	35.7	46.6	53.3	0	100	0	100
5	Amikacin(30)	64.3	35.7	93.3	6.6	100	0	100	0
6	Linezolid(30)	85.7	14.3	40	60	100	0	100	0
7	Cefoxitin(30)	85.7	14.3	46.6	53.3	100	0	0	100
8	Vancomycin(30)	_	_	100	0	100	0	100	0
9	Netilmicin(30)	100	0	66.6	33.3	100	0	100	0
10	Cotrimoxazole(25)	57.1	42.9	66.6	33.3	0	100	0	100
11	Cefotaxime(30)	57.1	42.9	53.3	46.6	100	0	100	0
12	Imipenem(10)	_	_	_	_	_	_	_	_

**Table 3:** Susceptibility pattern of commonly used antimicrobial agents in the treatment of Gram negative isolates causing neonatal septicaemia

Sn	Antibiotics (disk content in μg)	Gram-negativeorganisms													
		*Pseudomonas aeruginosa*		*Citrobacter freundii*		*Escherichia coli*		*Enterobacter aerogenes*		*Klebsiella pneumoniae*		*Acinetobacter spp*		*Proteus mirabilis*	
		S (%)	R (%)	S (%)	R (%)	S (%)	R (%)	S (%)	R (%)	S (%)	R (%)	S (%)	R (%)	S (%)	R (%)
1	Ampicillin(10)	0	100	0	100	25	75	14.3	85.7	10	90	0	100	0	100
2	Gentamicin(10)	50	50	100	0	50	50	57.1	42.8	30	70	0	100	0	100
3	Amoxycalvulinic acid(20/10)	33.3	66.6	100	0	87.5	12.5	28.5	71.4	60	40	62.5	37.5	0	100
4	Ceftriaxone(10)	50	50	0	100	62.5	37.5	57.1	42.8	30	70	50	50	0	100
5	Amikacin(30)	83.3	16.6	0	100	50	50	57.1	42.8	60	40	75	25	0	100
6	Linezolid(30)	50	50	100	0	100	0	57.1	42.8	60	40	0	100	100	0
7	Cefoxitin(30)	16.6	83.3	0	100	37.5	62.5	51.4	28.6	0	100	37.5	62.5	0	100
8	Vancomycin(30)	_	_	_	_	_	_	_	_	_	_	_	_	_	_
9	Netilmicin(30)	33.3	66.6	_	_	0	100	51.4	28.6	60	40	37.5	62.5	_	_
10	Cotrimoxazole(25)	50	50	0	100	37.5	62.5	28.6	51.4	20	80	50	50	0	100
11	Cefotaxime(30)	33.3	66.6	0	100	37.5	62.5	51.4	28.6	40	60	100	0	100	0
12	Imipenem(10)	83.3	16.6	0	100	62.5	37.5	85.7	14.3	90	10	100	0	100	0

### Factors associated with neonatal septicaemia

The risk factors for development of neonatal septicemia were maternal factors ie age of 25 years and above, parity of multiparous and grand multiparous, occupation (employed), education level (primary, secondary and tertiary), delivery mode of SVD and assisted delivery, ANC attendance of more than 4 time, UTI during pregnancy, premature rupture of membranes, Prolonged labor, Foul smelling liquor, urban residence. Neonatal factors ie birth weight ≥ 2500 g, Apgar score in the 1st minute and 5th minute ≥6, resuscitation and age category of between 72 h to 4wks. Of these factors, both early and late onset septicaemia neonatal septicemia were significantly associated with mother’s age of 25–35 and 36 years and above (*p*-value = 0.018 and *p*-value = 0.002 respectively), employed mothers (*p*-value = 0.001), tertiary-level of education (*p*-value = 0.023), SVD (*p*-value = 0.013), ANC (Antenantal care) attendance of more than 4 times (*p*-value = 0.028), UTI during pregnancy (*p*-value = 0.031), Premature rupture of membranes (*p*-value = 0.007), foul Smelling liquor (*p*-value = 0.033), Urban residence (*p*-value = 0.000), neonatal birth weight of ≥2500 g (*p*-value = 0.004), Apgarscore 1st min and 5th min of ≥6 with *p*-value = 0.000 and *p*-value = 0.016 respectively and resuscitation (*p*-value = 0.006) as shown in Table 4.

**Table 4:** Mother’s and neonatal’s characteristics associated with neonatal septicaemia

Variables	Odds Ratios	*p*-value	95% CI
Mothers factors			
Mothers’ age			
25-35 years	0.336	0.018	0.136–0.832
36 and above	0.225	0.002	0.086–0.591
Mothers’ parity			
Multiparous	0.751	0.522	0.313–1.802
Grand multiparous	1.262	0.609	0.516–3.092
Occupation			
Employed	0.281	0.001	0.132–0.600
Education level (none)			
Primary	0.868	0.829	0.240–3.135
Secondary	0.939	0.920	0.276–3.194
Tertiary	0.215	0.023	0.057–0.807
Mode of delivery			
SVD	3.281	0.013	1.290–8.344
Assisted delivery	2.007	0.126	0.822–4.900
ANC attendance			
More than 4 time	0.438	0.028	0.209–0.916
UTI during pregnancy			
Yes	2.252	0.031	1.078–4.705
Premature rupture of membranes			
Yes	2.810	0.007	1.320–5.982
Prolonged labor			
Yes	1.145	0.713	0.556–2.358
Foul Smelling liquor			
Yes	2.224	0.033	1.065–4.644
Residence			
Urban	0.247	0.000	0.114–0.539
Neonates factors			
Birth weight			
≥2500 g	3.385	0.004	1.470–7.791
Apgarscore 1st min			
≥6	0.173	0.000	0.068–0.435
Apgarscore 5th min			
≥6	0.360	0.016	0.157–0.823
Resuscitation			
Yes	0.355	0.006	0.168–0.747
Age category			
72 h-4wks	0.962	0.918	0.465–1.992

## Discussion

In this study, out off 122 paticipants, 56.6% were male and 43.4% female. The male were predominant which agrees with previous reports [25]. The blood culture positivity rate identified from neonats with symptoms of septicemia was 59.0%, this was a high blood culture-positivity rate as comparable to other findings [26]. The high prevalence could have been due to the fact that the study site (Kilembe mines hospital) was the only general hospital around Kasese town, most frequently receiving neonates with complications as well as complicated pregnancies than surrounding low level health facilities**.**

56.6% of the participants presented with early onset septicaemia and 43.4% with late onset septicaemia which agrees with the high prevalence reported by Islam [27, 28]. However, a study conducted at Mbarara regional referral hospital [29] indicated EOS of 24% (19/80 neonates) and LOS of 21.3 (7/80 neonates) with blood culture positivity of 32.5% (26/80 neonates). In our study, the positivity rates amongst neonates that presented with EOS and LOS were 41(56.9%) and 31(43.1%) respectively, this could have been due to infections asecending from the perineum of the mother or due to poor infection control during the delivery process. This was higher in male (55%) than female (44.4%) as also reported in other studies [30].

Of the 11 etiological agents identified, Group B *Strepococcus* (GBS) ie *Streptococcus agalactiae* (21%) was the most common amongst the neonates followed by *S. aureus* 19%, *Klebsiella pneumoniae* (14%), *(Escherichia coli* (11%), *Acinetobacter spp* (11%), *Enterobacter aerogenes* (10%), *Enterobacter aerogenes* (7%), *Citrobacter freundii* (1%), *Viridans streptococci* (1%), *Proteus mirabilis* (1%) and *Enterococcus.spp.*(1%). This was contrary to a study by Maimoona [31] who reported most common pathogens as *Klebsiella pneumoniae* (35%), followed by *Staphylococcus aureus* (24.1%). The difference could be due to difference in health care systems, population studied, diagnosis criteria and the case definition between the study sites [15].

Gram-negative and Gram-positive septicaemia was encountered in 56.9%(41) and 43.1%(31) of the culture positive cases in this study respectively, which was comparable to a study conducted by Gupta [32] and other studies where Gram-negative and Gram-positive organisms were responsible for 59 and 41% of the septicaemia cases, respectively as observed by Mugalu [4]. Gram negative organisms 41 (56.9%) were most implicated with neonatal septicaemia. This was also reported in the previous study [26]. However, this was contrary to a study conducted at Mulago hospital which indicated that Gram positive organisms were predominant (69.2%) [4].

Gram negative agents most responsible for neonatal septicaemia were *Klebsiella pneumoniae* 10(24.4%), *Escherichia coli* 8(19.5%) as reported in other findings [33], *Acinetobacter spp* 8(19.5%), *Enterobacter aerogenes* 7(17.1%), *Pseudomonas aeruginosa* 6(14.6), *Citrobacter freundii* 1(2.4%) and *Proteus mirabilis* 1(2.4%). *Klebsiella pneumoniae* was the predominant isolate (24.4%) among the Gram-negative pathogens which correlates with other findings [34]. However, this was contrary to a study which reported *Acinetobacter spp* (9.5%) as the most predominant Gram negative organism followed by *Klebsiella pneumoniae* (7.7%) [35]. The difference could have been due to changes in causative agents of neonatal septicaemia over time and may vary from place to place [36].

This study r that out of the 31(43.1%) Gram-positive organisms identified, majority of these were *Streptococcus agalactiae* 15(48.4%) as also reported by Nuorti [37] as the leading cause of invasive bacterial infections in newborn babies followed by *Staphylococcus aureus* 14 (45.2%), *Enterococcus.spp.* 1(3.2%) and *Viridans streptococci*1(3.2%).

From the analysis of drug susceptibility profiles according to the WHO recommended first and second-line antibiotics, our study showed that among Gram-negative isolates, majority of the isolates (92.9%) were resistant to ampicillin, Cefoxitin (76.7%), Cotrimoxazole (70.6%), Ceftriaxone (64.3%), Netilmicin (59.5%), Gentamicin (58.9%), Amikacin (53.3), Amoxyl/clavulinic acid (46.9%), Cefotaxime (45.5%) and Linezolid (33.3%). The least resistance was observed to imipenem (25.5%) as seen in other studies [38]. Among Gram-positive isolates, high resistance was observed to ampicillin (100%) similarly to a study by Mustafa [39], Gentamicin (80.9%), Ceftriaxone (72.3%), Cotrimoxazole (69.1%), Amoxyl/clavulinic acid (50.9%), Cefoxitin (41.9%), Amikacin (25.4%). There was no resistance of *Streptococcus agalactiae* to Vancomycin as also reported by other studies [40]. Overall, the least resistance was to Netilmicin (8.3%) followed by Linezolid (18.6%) and Cefotaxime (22.4%). Of the aminoglycosides used, amikacin (46.5%), exhibited a verge sensitivity over netilmicin (36.4%) and gentamicin (41.0%) against Gram negative organisms as observed in other studies [25].

Our study revealed that *Staphylococcus aureus* was more sensitive to netilmicin (100%) contraly to a study by Peterside [41] where ciprofloxacin was 90.9% effective. However, a study conducted by Lamba agrees to our study that Gram-positive isolates that include *Staphylococcus aureus* have good sensitivity to linezolid and vancomycin [42]. *Enterococcus.spp.*were equally sensitive to amoxycalvulinic acid, amikacin, linezolid, cefoxitin, vancomycin and netilmicin i.e., 100% dispite its resistance especially when the organisms are in large numbers as reported in other studies [43].

Of the Gram positive isolates, imipenem was found to be more effective to *Enterobacter aerogene, Pseudomonas aeruginosa* and *Acinetobacter spp*. This agrees with other findings [44]. Different studies [25] agree with the findings of our study indicating that imepenem had the overall best sensitivity (74.5%) among Gram-negative organisms.

In our study, more than two thirds of *Klebsiella pneumoniae* were resistant against third generation cephalosporins (ceftriaxone and cefotaxime) as also observated by Maramba-Lazarte [45] Prevalence of ESBL positivity was *40*, 25, 12.5 and 14.3% for *Klebsiella pneumoniae, Escherichi coli, Enterobacter aerogenes* and *Acinetobacter spp* respectively which could have been unrestricted use of antibiotics in heath units hence increase in the emergence of multidrug drug resistant organisms such as ESBL-producing orgamisms as repodted by other studies [46]. In this study, *Citrobacter freundii* was the most commonly reported as showing resistance (100%) to carbapenems (imipenem) which could be attributed to increased use of carbapenems even without prescriptions by clinicians. However this was different to other studies that showed *Klebsiella pneumoniae* as the most commonly resistant orgamism [47].

In our study, MRSA among *S*. *aureus* isolates *was* 79% (11/14) which was a high propotion as also reported in orther studies [48], which could have been due to transmission from the colonized maternal genital tract or from the labour ward after unhygienic obstetric practices. However this was relatively higher than what was reported by Eyob (72%), the difference could have been due to geographical differences ie variation in the frequency of the organisms within and between coutries, types of the specimen, laboratory procedures, study population, and study duration and differences in study design [49].

*Staphylococcus aureus* was more resistant to monobactam (ampicillin, amoxyl/calvulinic acid) and aminoglycoside (gentamicin). *S. auerus* is known to have remarkable feature to adapt antimicrobial pressures due to its genetic competence to develop antibiotic resistance genes from other strains.

*Klebsiella pneumoniae* also exhibits intrinsic resistance mechanisms with its chromosomal and plasmid encoded beta-lactam hydrolyzing enzymes (e.g. ESBLs) which could explain its greatest resistant to 2nd generation cephalosporins (cefoxitin) and aminopenicillin (ampicillin) in this study as mentioned in previous reports [50].

The most in effective antibiotic was ampicillin for both Gram negative and gram positive bacteria, this could have been due to selective resistance pressure expelled from the erratic use of the same antibiotics overtime. Similarly, consistent finding were also documented by Roja Rani Pallavali with a predominant resistance to ampicillin in both Gram positive and negative bacteria [51].

Maternal factors associated neonatal septicaemia found in this study were PROM and UTI during pregnancy. In this study, neonates born to mothers with these factors were more likely to develop septicaemia. Premature rupture of the membranes (PPROM) is a pregnancy complication where the sac (amniotic membrane) surrounding the baby breaks (ruptures) before week 37 of pregnancy. Mothers with early PROM and prolonged labor have increased chances of microorganisms ascending from the birth canal into the amniotic sac which causing fetal compromise as well as septicaemia during the neonatal period. This also explains the rationale for giving prophylactic antibiotic therapy to neonates born to mothers with a history of PROM during pregnancy which could increase chances of antimicrobial resistance. This is consistent with earlier studies conducted in different parts of the world [52, 53].. Urinary tract infections(UTI) occurs when bacteria enter the urinary tract through the urethra and begin to multiply in the bladder. When that happens, bacteria may take hold and grow into a full-blown infection in the urinary tract when a pregenant mother has UTI, the infection may frequently be transmitted to the baby in utero or during passage through the birth canal which may lead to neonatal septicaemia [54]. Spontaneous vaginal delivery (SVD) is a vaginal delivery that happens without using tools to help pull the baby out. This was associated with neonatal septicaemia in our study, here babies could have been exposed to maternal vaginal and fecal bacteria through per-vaginal examination during labour and delivery or as a result of bacteria being introduced into the cervical canal causing vertical transmission from infected mothers to the babies or chorioamnionitis caused by bacteremia that occurs when the amniotic sac breakes before birth [55, 56]. which was contrary to other studies that showed cesarean section was more associated with culture-positive cases [57]. This justifies the need for infection control practices and improving mother hygiene as reported by Ahmed [58]. In other studies, mothers who attended ANC late were not associated with neonatal septicaemia [4], this was contrary to our study where mothers who had ANC attendance of more than 4 times were more associated with neonatal septicaemia, though ANC utilization is vital in reducing the risk factors to neonatal septicaemia but that was not the case in our study and this could have been due to over crowding at ANC (that handled both ANC and postnatal services), use of only one toilet for all the out patients ie where mothers could have contracted infections and exposing their babies, using only one weiging scale for all the babies without decontamination or washing hands between babies. Foul Smelling liquor was also associated with neonatal septicaemia as similarly to other studies [59].

Our findings revealed that Apgarscore at the 1st and 5th minutes of ≥6 were highly associated with neonatal septicaemia because Apgar score at the 1st minute is associated with the hydrogen potential cord blood and intra partum depression, while the Apgar score at the 5th minute reflects the change in infants’ condition on the resuscitation performed which was not the case for a study conducted by Abdulhakeem [60]. In our study, majority of the neonates had adaptated well to extra uterine life without much stress experienced during labour. Association of Apgarscore ≥6 to septicaemia was likely to be due to the fact that when babies are in good health, a lot of people want to touch or carry the baby little knowing that they are exposing the baby to infections. Also unhygienic practices and not following guidelines by health workers when handling babies could expose neonates to infections [61]. It is largely understood that low birth weight infants are at high risk of developing septcaemia compared to normal birth weights. However, the present study indicated no significant association with this variables where we noted that neonatal birth weight of ≥2500 g and resuscitation of newborn babies were greatly associated with septicaemia. Similar findings were also observed in other previous studies in Ghana [61]. This could have been nosocomial infections or use of non-sterile equipments during resuscitation.

Mother’s occupation (employed) and urban residence also had an influence on neonatal septicaemia. This was contrary to other findings [60] where mother’s occupation status and urban residence were not found to be associated with neonatal septicaemia. This could have been attributed to nosocomial infections, poor infection prevention control measures or congested wards.

### Limitations

i. Our study size and study period were not enough to yield statistically precise estimates for most of the less common etiological agents for septicaemia.
ii. We might have underestimated the proportion of neonates with septicaemia because blood culture itself has a poor sensitivity especial with the small volumes of blood that were collected from the neonates [62].

## Conclusion

Neonatal septicaemia is becoming a life threatening emergency and it is evident from this study that Gram-negative organisms (*Klebsiella pneumoniae, Acinetobacter spp, Escheria coli*) and Gram positive organinsms (*Streptococcus agalactiae* and *Staphylococci aureus*) were the leading cause of neonatal septicaemia. The study of etiological profile, their antibiotic sensitivity pattern and risk factor of neonatal septicaemia plays a significant role. Definitive culture results takes at least 2–3 days leading to treatment delays. But with the use of improved bacteriological techniques such as BACTEC and BACT/ALERT, bacterial growth can be detected within 12–24 h. Appropriate use of antibiotic susceptibility surveillance programme along with good infection control practices and eloquent use of antibiotics to reduce the infection rate, ensure better therapeutic success, reduced drug resistance rates and prolong the efficacy of the available antimicrobials. Therefore, there is an urgent need for quick and accurate diagnostic tools for detection of systemic bacterial infections in neonates.

### Recommendations

Blood culture, surveillance of antimicrobial resistance are necessary and an antibiotic policy should be formulated in the hospital. Depending on the antibiotic sensitivity pattern of the isolates, antimicrobials can be used. Furthermore, we advise that health education be provided to the public on the dangers of undiscerning use of antibiotics, which is currently considered to be a menace in our society and which has been responsible for the ineffectiveness of the most commonly used antibiotics as observed in this study.

## Data Availability

The datasets used and/or analyzed during the current study is available from the corresponding author on reasonable request.

## Abbreviations

ANC: Antenatal care
API: Analytical profile index
ATCC: American type culture collection
BHI: Brain heart infusion
CLSI: Clinical & laboratory standards institute
DHO: District health officer
EOS: Early onset septicaemia
ESBL: Extended spectrum beta-lactamase
GBS: Group B *Strepococcus*
HMIS: Health management information systems
LOS: Late-onset septicaemia
MIC: Minimum inhibitory concentration
MRSA: Methicillin-resistant *Staphylococcus aureus*
NMR: Neonatal mortality rate
OPD: Outpatient department
PROM: Premature rupture of membranes
Spp: Species
SVD: Spontaneous vaginal delivery
UTI: Urinary tract infection
